# Dynamic variation of bacterial community assemblage and functional profiles during rice straw degradation

**DOI:** 10.3389/fmicb.2023.1173442

**Published:** 2023-04-14

**Authors:** Ruibo Sun, Xin Wang, Yousef Alhaj Hamoud, Mengxing Lu, Hiba Shaghaleh, Wenjie Zhang, Chaochun Zhang, Chao Ma

**Affiliations:** ^1^Anhui Province Key Laboratory of Farmland Ecological Conservation and Pollution Prevention, Research Centre of Phosphorus Efficient Utilization and Water Environment Protection Along the Yangtze River Economic Belt, College of Resources and Environment, Anhui Agricultural University, Hefei, China; ^2^Key Laboratory of JiangHuai Arable Land Resources Protection and Eco-restoration, Ministry of Natural Resources, Hefei, China; ^3^College of Hydrology and Water Resources, Hohai University, Nanjing, China; ^4^College of Environment, Hohai University, Nanjing, China; ^5^College of Resources and Environmental Sciences, National Academy of Agriculture Green Development, Key Laboratory of Plant-Soil Interactions, Ministry of Education, College of Resources and Environmental Sciences, China Agricultural University, Beijing, China

**Keywords:** straw degradation, bacterial community assembly, lignocellulosic decomposing bacteria, cellulose-decomposing bacteria, microbial interaction, straw returning

## Abstract

Bacteria is one of the most important drivers of straw degradation. However, the changes in bacterial community assemblage and straw-decomposing profiles during straw decomposition are not well understood. Based on cultivation-dependent and independent technologies, this study revealed that the “common species” greatly contributed to the dynamic variation of bacterial community during straw decomposition. Twenty-three functional strains involved in straw decomposition were isolated, but only seven were detected in the high-throughput sequencing data. The straw decomposers, including the isolated strains and the agents determined by functional prediction, constituted only 0.024% (on average) of the total bacterial community. The ecological network showed that most of the identified decomposers were self-existent without associations with other species. These results showed that during straw composition, community assembly might be greatly determined by the majority, but straw decomposition functions might be largely determined by the minority and emphasized the importance of the rare species in community-specific functions.

## Introduction

Straw is an important resource for agricultural production. It contains a high amount of nitrogen (N), phosphorus (P), potassium (K), and micronutrient elements. Thus, it is a potential substitute for chemical fertilizers (Sun et al., 2022a), and studies have found that straw returning efficiently improved soil structures and increased soil nutrient levels and crop yield (Sun et al., 2015; Chen et al., 2017, 2022). However, the direct straw return may also result in environmental and production risks in agricultural systems, such as stimulating CH_4_ emission and aggravating plant pest infestation (Li et al., 2018; Kerdraon et al., 2019). These issues are closely associated with the low rate of straw decomposition. Thus, accelerating straw decomposition is of great importance for the eco-friendly utilization of straw.

Microbes are the primary drivers for straw decomposition. Microbes decompose the components of straw by producing enzymes such as glucosidase, cellobiohydrolase, and xylanase (Reddy et al., 2013; Zhou et al., 2021). In addition, some microbes, such as some mycelial fungi, can physically damage the straw through the massive growth of hyphae. However, straw is made up of various complex organic substances. Besides cellulose, hemicellulose, and lignin, types of proteins, lipophilic compounds, starches, low-molecular carbohydrates, and other organic compounds have been identified in straws (Van Hung et al., 2020; Rosado et al., 2022). Thus, straw decomposition is associated with diverse microbes targeting the different components, and the microbial communities vary greatly during degradation. For example, Orlova et al. (2020) found that bacterial diversity increased during the process of oats straw decomposition, and Proteobacteria, Firmicutes, and Bacteroidetes dominated in the early stage (3rd day), while Actinobacteria dominated in the late stage (161st day). A study in a paddy field found that bacterial diversity showed a bell-shaped curve during the 2 years. Straw decomposition was dominated by copiotrophic phylotypes, such as Bacilli and Flavobacteriia, in the early stages and evolved to be dominated by oligotrophic phylotypes, such as Acidobacteria, Anaerolineae, Deltaproteobacteria, Saccharibacteria, and Sphingobacteriia, in the later stages (Wang et al., 2022).

As cellulose, hemicellulose, and lignin are straw’s main components, accounting for about 75% of rice straw’s biomass, and these biopolymers are normally decomposed slowly, the microbes targeting these components have attracted more attention than other functional types. These cellulose/lignin-decomposing microbes primarily decompose straw by producing degrading enzymes, including cellulases, hemicellulases, and lignin-degrading enzymes (Bayer et al., 2006; Floudas, 2021). Enhancing the function of these microbes is the primary pathway to accelerate straw decomposition. Many approaches have been developed to enhance microbial activities to accelerate the speed of straw decomposition, such as adding trace elements (Jin et al., 2022) or nanomaterials (Qiu et al., 2019) and improving planting strategies (Zhou et al., 2021). In addition, the construction of microbial consortiums with high straw degradation capacity is also considered a potential straw treatment method (Gong et al., 2020; Sarma et al., 2022). However, most of the present studies were performed in controlled systems; regulating microflora to enhance straw decomposition is still a great challenge in practical production. The microbial community is a complex consortium; the members are closely associated with each other (Liu et al., 2019), so it is difficult to regulate the functional groups involved in straw decomposition without considering other groups. Although many straw-decomposing strains have been isolated, and they show high capacity in straw decomposition in controlled experiments (Gupta et al., 2012; Lestari et al., 2020; Sun S. et al., 2020), how to make them perform the functions in practical systems is still a difficult problem that had not been solved, as the isolated straw-decomposing strains are difficult to colonize in the natural environments due to the great effects of environment filter and the strong competition from the indigenous residents (Sun R. et al., 2020).

Straw-decomposing microbes are widely distributed in types of ecosystems and habitats, especially in agricultural soils, but they are not alone; their growth, multiply, and functions are greatly impacted by other species (Bayer et al., 2006). Thus, understanding the associations between straw-decomposing agents and other species is crucial for regulating microbial community functions. Although many studies have revealed the variation of microbial communities during straw decomposition, we still know little about the dynamic changes of functional agents in the whole community and the relationships between the functional agents and other microbial types. This study explores the dynamic variation of bacterial community assemblage and straw-decomposing agents based on cultivation-dependent and independent techniques and the correlations between functional agents and other microbial groups. The results from this study will give new information about the microbial mechanisms of straw degradation.

## Materials and methods

### Experiment design and soil sampling

A field experiment was established in June 2019 in the National Innovative and High Technology Agricultural Park of Anhui Agricultural University, Anhui Province, China (N 31°93′, E 117°20′). The soil type is Luvisols.

The field is under rice-wheat rotation. The basic properties of the studies soil were as follows, pH 6.15, soil organic matter (SOM) 11.40 g/kg, total nitrogen (TN) 0.48 g/kg, alkali-hydrolyzable nitrogen (AHN) 45.80 g/kg, available phosphorus (AP) 6.28 mg/kg, and available potassium (AK) 0.16 g/kg.

After desiccating by a dryer, wheat straw was cut into pieces (1–3 cm) and then put into nylon bags (15 cm × 20 cm, mesh size was 0.075 mm). Each bag contained 20 g wheat straw. Before rice transplanting in June 2019, the nylon bags were embedded into soils with a depth of 5–20 cm. The experiment contained three replicate plots (12 m^2^), and each plot contained 12 nylon bags. After treatment, rice was transplanted, and the field was managed as the local strategy.

Three nylon bags were removed from each plot at 7, 15, 60, and 120 days. Each sample was divided into two parts. One was stored at 4°C to isolate cellulose- and lignin-decomposing bacteria and the other was stored at −80°C for DNA extraction.

### Isolation of cellulose- and lignin-decomposing bacteria

An enrichment culture was performed before the strain isolation. A 0.5 g sample was added to a 100 mL enrichment medium. The medium for cellulose-decomposing bacteria enrichment contained sodium carboxymethylcellulose (CMC-Na) 20 g/L, microcrystalline cellulose 5 g/L, cellulose powder 5 g/L, K_2_HPO_4_ 1 g/L, KNO_3_ 1 g/L, MgSO_4_·7H_2_O 0.2 g/L, CuCl_2_·2H_2_O 0.1 g/L, and FeCl_3_ 0.02 g/L. The medium for lignin-decomposing bacteria enrichment contained sodium lignin sulfonate 3 g/L, NH_4_SO_4_ 2 g/L, K_2_HPO_4_ 1 g/L, MgSO_4_·7H_2_O 0.2 g/L, CaCl_2_ 0.1 g/L, MnSO_4_ 0.02 g/L, KH_2_PO_4_ 1 g, FeSO_4_ 0.05 g/L. The cellulose- and lignin-decomposing bacteria enrichment cultures were incubated at 36°C (180 rpm) and 28°C (200 rpm) for 3 days, respectively.

The functional strains were isolated using a dilution plate method (Fathallh Eida et al., 2012). 10 mL of enrichment culture was added into 90 mL sterile distilled water, and the mixture was then shaken at 150 rpm for 30 min at room temperature. Ten-fold serial dilutions were then prepared in sterilized distilled water. One-hundred microliters from the 10^−6^, 10^−7^, and 10^−8^ dilutions were spread on the isolation medium. The medium for cellulose-decomposing bacteria isolation contained CMC-Na 15 g/L, NH_4_NO_3_ 1 g/L, yeast extract 1 g/L, MgSO_4_·7H_2_O 0.5 g/L, KH_2_PO_4_ 1 g/L, and agar 15 g/L, and that for lignin-decomposing bacteria isolation contained sodium lignosulphonate 3 g/L, NH_4_SO_4_ 1 g/L, KH2PO4 1 g/L, MgSO_4_·7H_2_O 0.2 g/L, CaCl_2_ 0.1 g/L, MnSO_4_ 0.02 g/L, MnSO_4_ 0.02 g/L, KH_2_PO_4_ 1 g, FeSO_4_ 0.05 g/L, and agar 15 g/L. The cellulose- and lignin-decomposing bacteria isolation plates were incubated at 36°C and 28°C for 5 days, respectively.

After incubation, the cellulose- and lignin-decomposing colonies were isolated and transferred to CMC-Na Congo red medium (CMC-Na 1.88 g/L, K_2_HPO_4_ 0.5 g/L, MgSO_4_ 0.25 g/L, gelatin 2 g/L, Congo red 0.2 g/L, agar 15 g/L) and aniline blue medium (yeast extract powder 10 g/L, glucose 10 g/L, aniline blue 0.1 g/L, agar 15 g/L) respectively to confirm the decomposition capacity. All the colonies containing decomposition capacity were transferred to the LB plate to obtain a pure culture.

### PCR amplification and sequencing of isolated bacteria

Colony PCR was used for the amplification of bacterial 16S rRNA using the primer sets of 27F (5′- GAGTTTGATCMTGGCTCAG-3′) and 1492R (5′-TACGGYTACCTTGTTACGACTT-3′). The amplification was performed in a 50-μL system with the thermal cycle of the initial denaturation step at 94°C for 2 min, 30 cycles with denaturation at 98°C for 10 s, annealing at 51°C for 30 s, and extension at 72°C for the 90 s, then a final extension at 72°C for 5 min. The 50-μL PCR system contained 25 μL PCR premix (Ex TaqTM; Takara, Shiga, Japan), 1 μL (10 μM) of the forward and reverse primers, and 23 μL sterile double-distilled water. After being purified using a QIAquick PCR Purification Kit (Qiagen, Hilden, Germany), the PCR products were sequenced using a 3730xl DNA analyzer (Applied Biosystems).

### Total DNA extraction, PCR, and high-throughput sequencing

The bacterial community was detected as described in previous studies (Sun et al., 2022b,c). In short, total DNA was extracted from a 0.5 g sample using a FastDNA SPIN Kit (MP Biomedicals, Santa Ana, CA, United States). Before DNA extraction, the samples were frozen using liquid nitrogen and then grinded thoroughly. Primer sets 515F/907R targeting the V4-V5 region of 16S rRNA gene were used for PCR amplification, which was performed in a 25-μL system containing 12.5 μL PCR premix (Ex TaqTM; Takara, Shiga, Japan), 0.5 μL (10 μM) of each primer, 0.5 μL DNA template (20 ng), and 11 μL sterile double-distilled water with the thermocycler of an initial denaturation at 94°C for 10 min, 30 cycles of denaturation at 94°C for 30 s, annealing at 55°C/56°C (16S rRNA/ITS) for 45 s, and extension at 72°C for 1 min, followed by a final extension at 72°C for 10 min. After purification, the PCR products were sequenced using the Illumina HiSeq2000 platform (Illumina, San Diego, CA, United States).

### Bioinformatic analysis of the high-throughput sequencing data

Bioinformatic analysis was performed primally using the VSEARCH package (version 2.21.1) (Rognes et al., 2016) according to the pipelines described in a previous study (Sun et al., 2023). The adept and primer sequences were cut using Cutadapt (version 4.2) (Martin, 2011). Then the paired-end reads were merged, and low-quality reads (length < 150 bp, expected errors per base >0.001) were filtered. After removing chimeric reads using the UCHIME algorithm (Edgar et al., 2011), the clean reads were denoised using the UNOISE algorithm (version 3), and zero-radius operational taxonomic units (zOTUs) were generated. Taxonomic assignment of each zOTU was performed using SINTAX (Edgar, 2016) based on the Silva database (version 138). The unidentified zOUTs were removed, and the zOTU table was rarified to 19,000 reads per sample for downstream analysis.

The V4-V5 region of the 16S rRNA sequences of the isolated strains was extracted using Cutadapt (version 4.2) (Martin, 2011). Then the trimmed sequences were clustered with the sequence of each zOTU at the similarity of 100% to find which zOTU they matched.

FAPROTAX (Louca et al., 2016) was used for the functional prediction of the bacterial community, and the function categories involved in straw decomposition were selected for further analysis.

### Statistical analysis

R (version 4.0.2) was used for statistical analysis using the relevant libraries (Sun et al., 2019, 2021). The Kruskal–Wallis rank sum test was used to check the significance of the difference between treatments using the “dplyr” library. Principal coordinate analysis (PCoA) based on Bray–Curtis distance was performed using the “vegan” library to show the variation of bacterial community among treatments. The abundance-based beta-null model was used to determine the role of niche and neutral processes in shaping bacterial communities during straw decomposition (Tucker et al., 2016). The bacterial ecological network was inferred using SPIEC-EASI (Sparse InversE Covariance Estimation for Ecological Association Inference) (Kurtz et al., 2015). Only the zOTUs detected in more than 75% of the samples were incorporated in the construction of the network to strengthen its reliability of the network (Sun R. et al., 2020). The characteristics of the network were calculated using the “igraph” library. The beta-null deviation was calculated to infer the changes in deterministic and stochastic processes in shaping bacterial community assembly (Tucker et al., 2016).

## Results

### Variation of bacterial community during rice straw degradation

**Figure 1:**
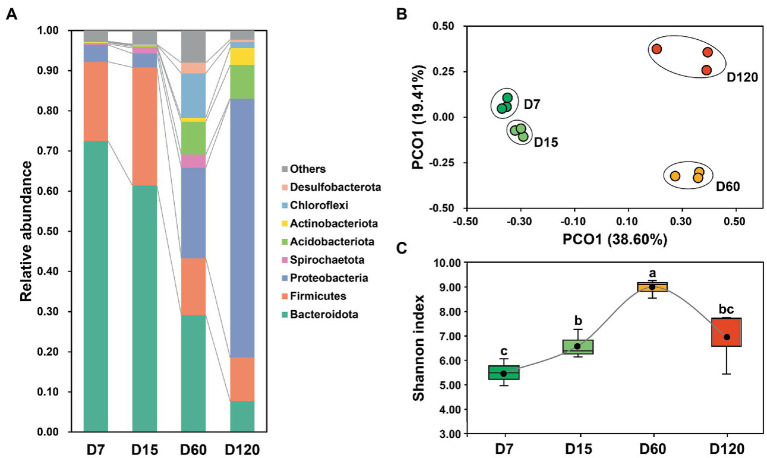
Variation of bacterial community composition during experimental session **(A)**. A PCoA plot of the bacterial community in different stage **(B)**. Dynamic variation of bacterial diversity during experimental session **(C)**. Different letters indicate significant differences as checked by the Kruskal-Wallis rank sum test (*p* < 0.05).

The bacterial community in the studied samples was dominant by Bacteroidota, Firmicutes, Proteobacteria, Spirochaetota, Acidobacteriota, Actinobacteria, Chloroflexi, and Desulfobacterota, accounting for 95.89% of the total reads (Figure 1A). The composition of the bacterial community varied obviously during rice straw degradation. The dominance of Bacteroidota and Firmicutes decreased during the experiment, while the relative abundance of Proteobacteria and Acidobacteriota was significantly higher in the late stage (60 and 120 days) than in the early stage (7 and 15 days) (Figure 1A). The dynamic variation of the bacterial community was illustrated clearly in the 2D PCoA plot (Figure 1B), in which the bacterial community from different stages separated from each other. The bacterial community from D60 and D120 was far separated from that from D7 and D15, showing the great variation of the bacterial community after 15 days (Figure 1B).

The Shannon index was calculated to explore the variation of bacterial diversity during the experiment. During the 120 days, bacterial diversity showed a bell-shaped curve (Figure 1C). Bacterial diversity increased from D7 to D60, then decreased from D60 to D120.

**Figure 2:**
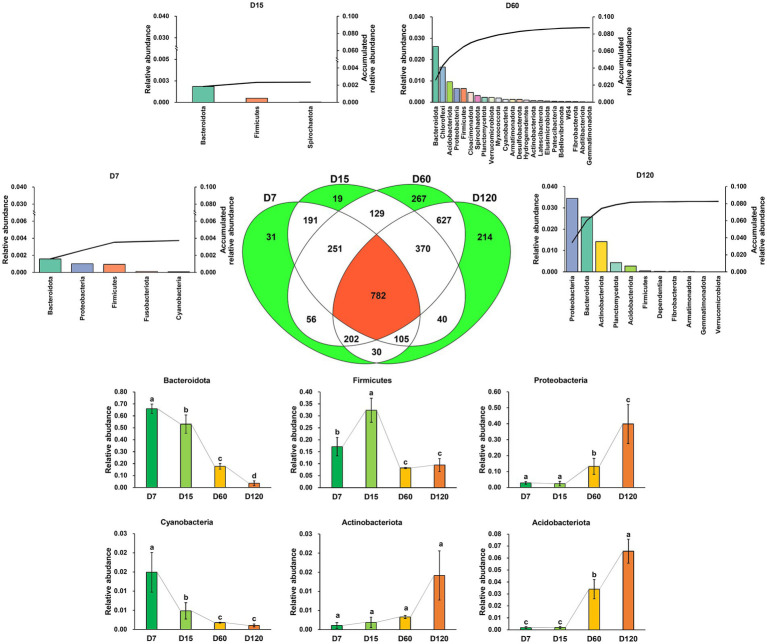
A Venn diagram showing the zOTU distribution in different stages. The bar charts denoted with stage name indicate the taxonomic composition and their accumulated relative abundance of the unique zOTUs in the stage. The bar charts at the bottom of the figure showed the variation of the dominant phyla of the shared zOTUs. Different letters indicate significant differences as checked by the Kruskal-Wallis rank sum test (*p* < 0.05).

In total, 3,314 zOTUs were detected in all the samples, and 782 zOTUs were shared in all samples. The number of unique zOTUs in D7, D15, D60, and D120 was 30, 19, 267, and 214, respectively (Figure 2). The unique zOTUs in each sample were rare species in the whole community; the total relative abundance of unique zOTUs in D7, D15, D60, and D120 was 0.37, 0.24, 8.74, and 8.26%, respectively. Differently, the community was dominated by the shared zOTUs, which accounted for 88.32, 85.01, 47.11, and 60.17% of the community in relative abundance for D7, D15, D60, and D120, respectively. Most of the shared OTUs were assigned as Bacteroidota, Firmicutes, Proteobacteria, Cyanobacteria, Actinobacteriota, and Acidobacteriota (Figure 2), but the taxonomic composition of the bacterial community varied greatly during the experiment. The relative abundance of shared Bacteroidota and Cyanobacteria decreased with time, while that of shared Proteobacteria, Actinobacteriota, and Acidobacteriota increased with time.

### Bacterial community assembly during straw decomposition

**Figure 3:**
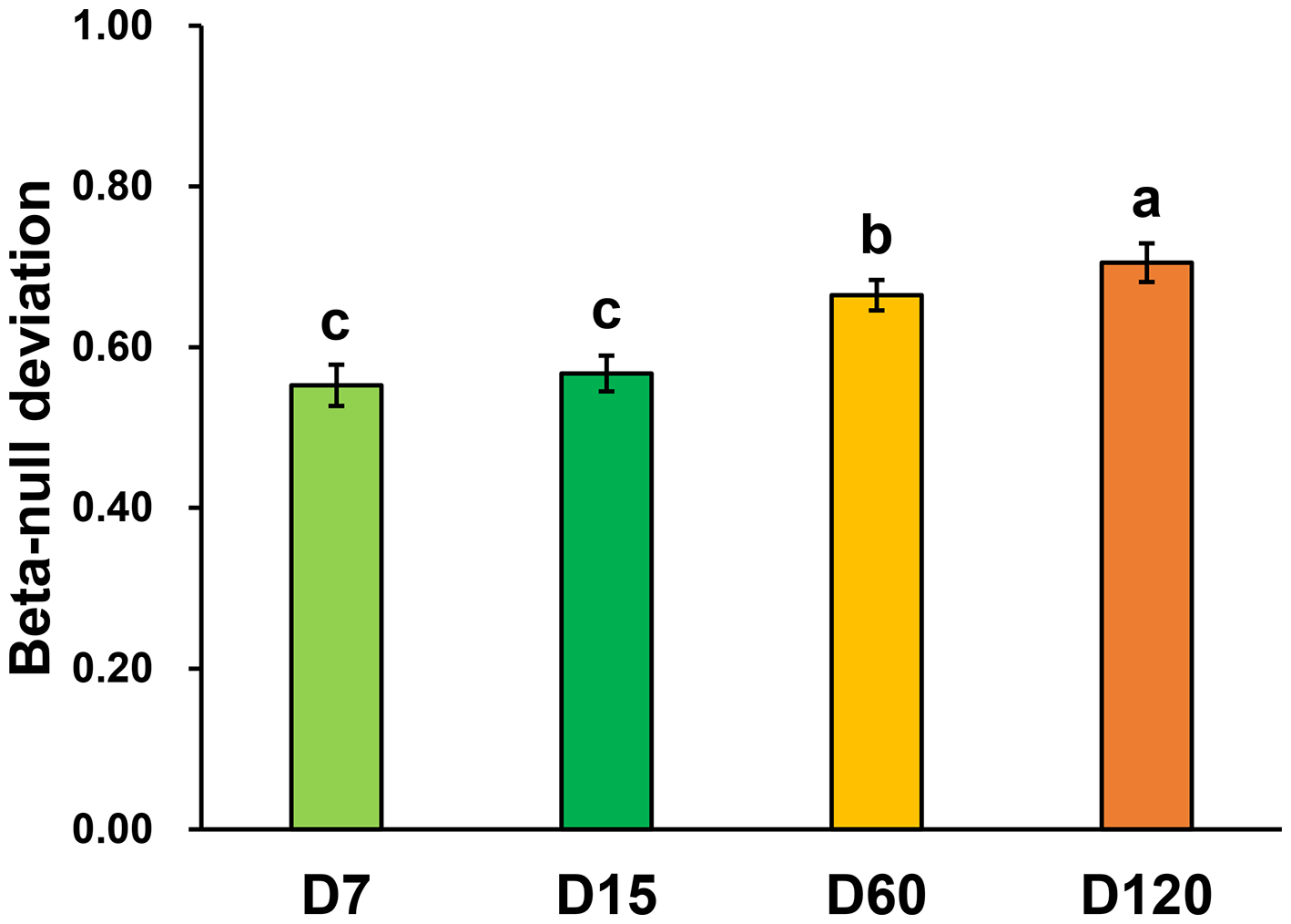
Abundance-based beta-null deviation for bacterial communities based on the Bray–Curtis distance. Different letters indicate significant differences as checked by the Kruskal-Wallis rank sum test (*p* < 0.05).

By calculating the beta-null deviation of the bacterial community in each stage, the contribution of niche and neutral processes in shaping the bacterial community was inferred. The beta-null deviations in all the samples were positive, showing the bacterial community as less similar than expected by chance and reflecting communities structured by non-neutral assembly mechanisms. Overall, the beta-null deviation of the bacterial community was increased during the experimental session (Figure 3). Beta-null deviations in D7 and D15 were significantly lower than in D60 and D120, and the biggest beta-null deviation was observed in D120. This result showed that the contribution of niche processes increased in structuring bacterial community during straw decomposition.

### Variation of functional profiles involved in straw decomposition

**Figure 4:**
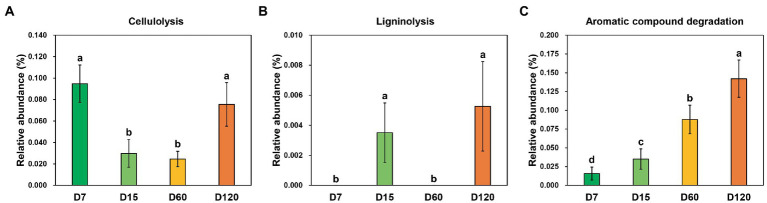
Variation of the functional profiles involved in straw decomposition determined by functional prediction. **(A)** Cellulolysis function. **(B)** Ligninolysis function. **(C)** Aromatic compound degradation. Different letters indicate significant differences as checked by the Kruskal-Wallis rank sum test (*p* < 0.05).

Three functional categories involved in straw decomposition were detected through functional prediction, including cellulolysis, ligninolysis, and aromatic compound degradation. The dynamic variation of the relative abundance of zOTUs involved in cellulolysis showed an inverted bell curve during the experiment, which was significantly higher in D7 and D120 than in D15 and D60 (Figure 4A). The species involved in ligninolysis were only detected in the samples from D15 and D120, and their relative abundance was quite low (Figure 4B). Differently, the species with aromatic compound degradation capacity increased with experiment time (Figure 4C).

Twenty-three strains with the potential of straw decomposition were isolated, including 15 cellulose-decomposing strains and 8 lignin-decomposing strains. By comparing the sequence information of 16S rRNA, seven strains were detected in the community determined by high-throughput sequencing, which matched 5 zOTUs in the community (Table 1). The relative abundance of these functional strains was low in the whole bacterial community, and they varied during the experimental session (Table 1). The relative abundance of cellulose-decomposing zOTU increased with experimental time, while the changes of zOTUs involved in lignin decomposition were various (Table 1).

**Table 1:** Changes in relative abundance (%) of cellulose and lignin degrading bacteria during straw degradation.

Function	OTU ID	D7	D15	D60	D120	Taxonomy
Cellulose decomposition	zOTU1413	NA	0.0018	0.0035	0.0228	g_*Stenotrophomonas*
Lignin decomposition	zOTU1386	0.0070	0.0035	0.0105	0.0105	s_*Bacillus aryabhattai*
	zOTU356	0.0596	0.0070	0.0228	0.0123	f_Enterobacteriaceae
	zOTU1824	0.0123	NA	NA	NA	s_*Escherichia coli*
	zOTU1049	0.0088	0.0579	0.0035	0.0088	g_*Pseudomonas*

NA indicates not detected.

f, g, and s indicate family, genus, and species level, respectively.

### Associations between straw decomposing agents and other bacterial species

**Figure 5:**
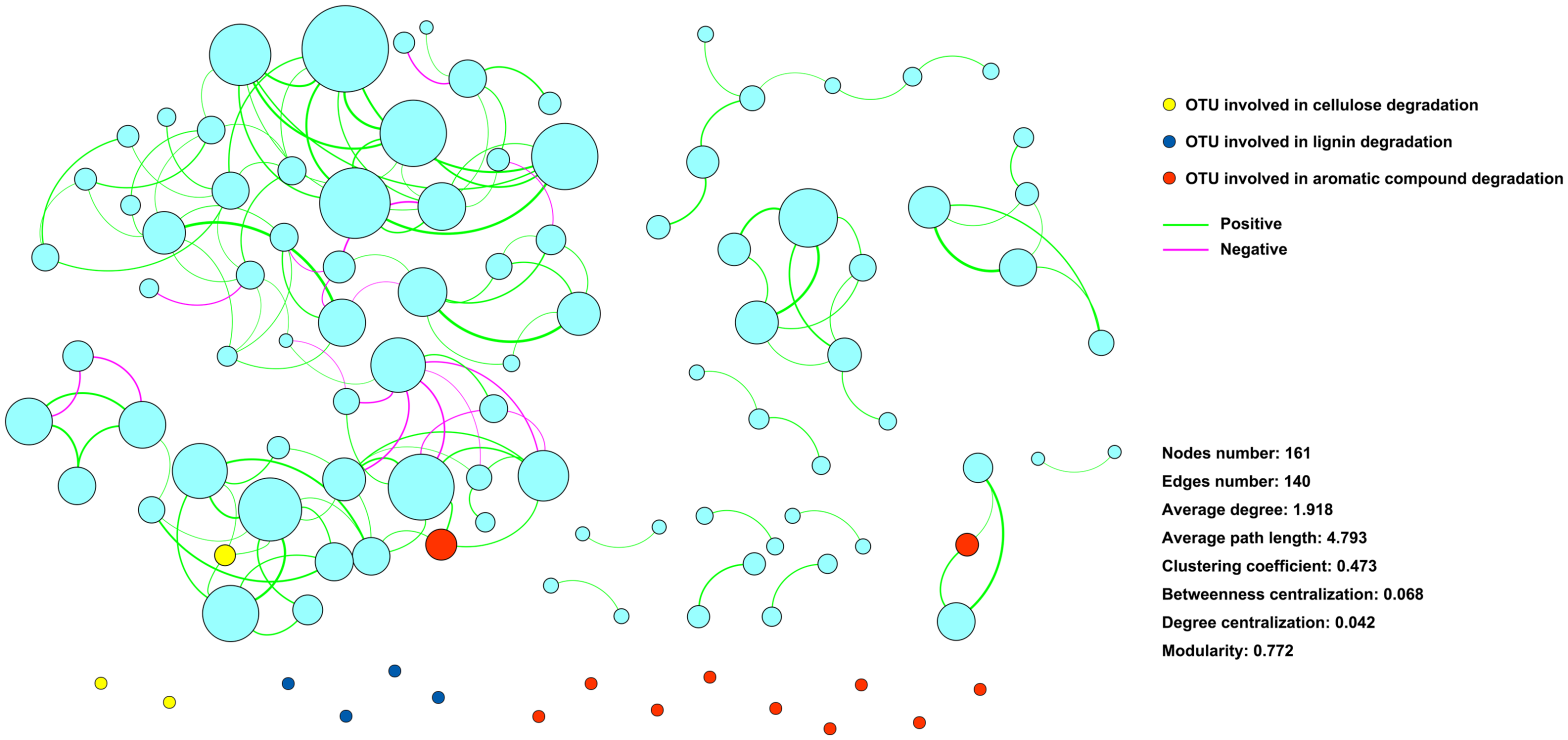
Ecological network showing the associations among community components. Nodes represent zOTUs, and the size of the node indicates the relative abundance of the zOTU. Nodes colored with yellow, blue, and red indicate zOTUs involved in cellulose, lignin, and aromatic compound degradation, respectively. Edges indicate associates between zOTUs.

An ecological network with 161 zOTUs (nodes) and 140 edges was constructed to infer the associations between the community members (Figure 4). Eighteen zOTUs with the capacity for cellulose, lignin, and aromatic compound degradation were incorporated into the ecological network. Only 3 zOTUs were associated with other zOTUs, and the other 15 zOTUs had no association with other zOTUs (Figure 5).

## Discussion

Straw is a carbon-rich resource containing valuable nutrients for plant growth. Thus, the effective utilization of straw is of great importance for agricultural production and environmental protection, especially for large agricultural countries such as China (Chen et al., 2022). However, the low speed of decomposition strongly restricts the effects of the direct return of straw on agricultural production. As the key driver for straw decomposition, microbes are the potential solution for this issue. Understanding the mechanisms of microbial community assembly and function dynamics is the premise for microbial community regulation. The variation of microbial composition and diversity during straw decomposition has been widely reported (Orlova et al., 2020; Zhong et al., 2020; Zhang et al., 2021). In this study, through the beta-null model, we further demonstrated that the contribution of the niche process was increased during the decomposition (Figure 3). This may be partly associated with the changes in competitive intensity between community members. In the early stage, all the microbes have almost equal opportunity (without regard to their difference in population) to decompose straw. In addition, the competition between community members is weak due to the high content of readily decomposable carbon fractions in the early stage (Preston et al., 2009). Thus, the community in the early stage was more likely randomly assembled. As decomposition goes on, recalcitrant carbon fractions, such as cellulose, lignin, waxes, and tannins, gradually accumulate (Wang et al., 2022). As a result, the effect of substrate limitation becomes stronger and stronger, and the microbes targeting these substrate gains a competitive advantage and show an increase in relative abundance (Orlova et al., 2020). We also observed the gradual enrichment of aromatic compound-decomposing bacteria during the experiment (Figure 4C). Thus, the contribution of the niche processes in shaping bacterial communities increased.

The role of abundant and rare species in community assembly, diversity, and ecological functions is a key topic in microbial ecology research (Magurran and Henderson, 2003; Zhang et al., 2020, 2022). The contribution of the species with different abundance in community variation and straw decomposition is little known. The results from the present study showed that the variation of the bacterial community was primarily associated with the “common species” (Figure 2), which was the species that existed during the whole process of straw decomposition and was termed as “core microbiome” too. The core microbiome is important for community function and microbial associations (Neu et al., 2021). However, in the present study, within the core bacterial community, the species directly associated with straw decomposition, including cellulolysis and aromatic compound degradation, was not dominant in relative abundance. The average relative abundance of zOTUs involved in cellulolysis and aromatic compound degradation was 0.056 and 0.048%, respectively (Supplementary Table S1), indicating that most common species were not directly associated with straw decomposition. Even with the respect of the whole community, the species directly associated with straw decomposition were also a minority (Figure 3). These results indicate that the decomposition of straw may largely depend on minority species. Due to the high functional redundancy of microbial communities (Allison and Martiny, 2008), microbial communities’ whole potential functional profiles were more stable than the taxonomic profiles. For example, Bao et al. (2020) found that the functional composition of the microbial community associated with straw decomposition was more stable than taxonomic composition. However, this conclusion was obtained based on the broad functional profiles of the microbial community. For some specific functions, the conclusion may be different. By growing many bacterial communities under the same environmental conditions, Rivett and Bell (2018) revealed the negative statistical interactions among abundant phylotypes drive variation in broad functional measures (respiration, metabolic potential, cell yield), whereas positive interactions between rare phylotypes influence narrow functional measures (the capacity of the communities to degrade specific substrates).

In contrast, rare phylotypes positively interacted with narrow functional measures (the capacity to degrade specific substrates), demonstrating that unique components of complex communities are associated with different ecosystem functioning. In general, unique species are rare in the community. Thus, the specific function of the community may be largely determined by the rare species, which also indicates the low redundancy of the specific functions.

The low abundance of species directly involved in straw decomposition may be one important reason for the low speed of straw decomposition. However, low abundance does not mean low activity. Studies have found that low-abundance species display disproportionately high activity for specific functions (Pascoal et al., 2020). For example, the high activity of sulfate reduction was driven by the rare biosphere microorganism in a peatland (Pester et al., 2010). The rare biosphere bacteria played a predominant role in phenanthrene degradation (Sauret et al., 2014), as some low-abundance microorganisms can be highly transcriptionally active without growth (Hausmann et al., 2019). Thus, how to increase the activity of the rare species involved in straw decomposition is a significant issue for the acceleration of straw decomposition.

The interactions between microbial community components affect microbial functions (Liu et al., 2019). Our previous study revealed that the organic phosphorus-mineralizing microbial taxa were regulated by the keystone taxa of the microbial community (Chen et al., 2020). The present study showed that few of the identified straw-decomposing species were associated with other species. In contrast, most of the functional species were independent in the ecological network (Figure 5), showing the diversiform survival types of the functional species. This may be important for sustaining microbial straw-decomposing capacity in complex and dynamic environments. Understanding the role of these functional species in straw decomposition would provide crucial information for regulating microbial straw-decomposing functions.

Although many achievements in microbial community assembly and functions have been obtained during the past years, especially from devising high-throughput sequencing technologies, we may still be far from the truth, as the sequencing technologies contain large biases, and most of the microorganisms are still unculturable. In the present study, 23 functional strains were isolated. Still, only seven were detected in the high-throughput sequencing data, indicating that many species had been omitted in the sequencing data. The low coverage of the primers may be the primary issue, and using PCR-bias-free technologies, such as metagenomic sequencing, may provide more accurate results (Sun et al., 2021). In addition, due to the limitations of microbial isolation and culture technologies, types of functional strains are still unculturable. As a result, many functional microbes are omitted and the role of the functional microbes in community assemblage and ecological functions may be underestimated. However, the great challenge of strain isolation and culture may be the stumbling block lasting for a long time. What’s more, straw decomposition is associated with types of microbes, especially fungi (Krishna and Mohan, 2017; Bani et al., 2018), thus understanding the associations between different microbial types is also important.

## Conclusion

Understanding the mechanisms of microbial community assembly and functional profiles is vital for managing straw decomposition by regulating microbial communities. This study revealed the dynamic variations of bacterial community assembly and straw-decomposing function during straw decomposition. Results showed that the variations of bacterial community greatly resulted from the changes of “common species,” and the contribution of niche process in structuring bacterial community increased during the decomposition. The relative abundance of the functional bacterial agents involved in straw decomposition was low in the whole bacterial community, and only a few of them were associated with other species, indicating that the rare species may greatly determine the functional profiles involved in straw decomposition and most of the functional agents were self-existent.

## Data availability statement

The datasets presented in this study can be found in online repositories. The names of the repository/repositories and accession number(s) can be found at: https://www.ebi.ac.uk/ena, PRJEB60739.

## Author contributions

RS, CZ, and CM designed the experiment and wrote the manuscript. XW and ML provided experimental materials and directed the experiment. XW, ML, and WZ performed the laboratorial measurements. RS performed the data analysis. YH and HS performed language edition and correction. All authors contributed to the article and approved the submitted version.

## Funding

This work was supported by the Key Science and Technology Project of Anhui Province (202103a06020012), the National Natural Science Foundation of China (32071628), and the Colleges and Universities Science Foundation of Anhui Province (KJ2021ZD0009).

## Conflict of interest

The authors declare that the research was conducted in the absence of any commercial or financial relationships that could be construed as a potential conflict of interest.

## Publisher’s note

All claims expressed in this article are solely those of the authors and do not necessarily represent those of their affiliated organizations, or those of the publisher, the editors and the reviewers. Any product that may be evaluated in this article, or claim that may be made by its manufacturer, is not guaranteed or endorsed by the publisher.
