# Supplementary material for: Dynamic variation of bacterial community assemblage and functional profiles during rice straw degradation
Source: Front Microbiol. 2023 Apr 14;14:1173442. doi: 10.3389/fmicb.2023.1173442 (PMC10140369; doi:10.3389/fmicb.2023.1173442)
Supplement: Supplementary file 1 [file Table_1.DOCX]

Table S1 The functional potential of the zOTUs shared in all samples

| **Function category** | **D7** | **D15** | **D60** | **D120** |
| --- | --- | --- | --- | --- |
| Animal parasites or symbionts | 40.632±8.435 | 16.596±2.726 | 1.981±1.457 | 2.861±3.322 |
| Chemoheterotrophy | 10.314±2.072 | 6.835±3.206 | 3.789±0.549 | 24.937±15.623 |
| Aerobic chemoheterotrophy | 0.614±0.571 | 1.346±1.404 | 2.054±0.613 | 24.544±15.558 |
| Fermentation | 9.605±2.337 | 5.472±1.874 | 1.739±0.257 | 0.304±0.144 |
| Human pathogens all | 0.258±0.061 | 0.204±0.124 | 1.688±1.271 | 0.511±0.294 |
| Human associated | 0.258±0.061 | 0.204±0.124 | 1.688±1.271 | 0.511±0.294 |
| Human pathogens pneumonia | 0.089±0.043 | 0.163±0.127 | 1.651±1.259 | 0.475±0.270 |
| Nonphotosynthetic cyanobacteria | 1.493±0.519 | 0.486±0.213 | 0.177±0.010 | 0.105±0.038 |
| Nitrogen fixation | 0.154±0.162 | 0.684±0.904 | 0.114±0.040 | 1.102±0.506 |
| Nitrate reduction | 0.374±0.166 | 0.744±0.911 | 0.546±0.340 | 0.179±0.082 |
| Nitrate respiration | 0.184±0.049 | 0.065±0.019 | 0.475±0.284 | 0.158±0.068 |
| Nitrogen respiration | 0.184±0.049 | 0.065±0.019 | 0.475±0.284 | 0.158±0.068 |
| Ureolysis | 0.153±0.160 | 0.668±0.908 | 0.030±0.029 | 0.005±0.004 |
| Plant pathogen | 0.654±0.379 | 0.051±0.039 | 0.016±0.015 | 0.012±0.009 |
| Nitrite respiration | 0.172±0.046 | 0.047±0.009 | 0.167±0.063 | 0.049±0.024 |
| Dark hydrogen oxidation | 0.011±0.007 | 0.009±0.009 | 0.374±0.300 | 0.019±0.020 |
| Nitrate ammonification | 0.168±0.049 | 0.040±0.009 | 0.037±0.026 | 0.035±0.025 |
| Nitrite ammonification | 0.168±0.049 | 0.040±0.009 | 0.037±0.026 | 0.035±0.025 |
| Human gut | 0.168±0.049 | 0.040±0.009 | 0.037±0.026 | 0.035±0.025 |
| Mammal gut | 0.168±0.049 | 0.040±0.009 | 0.037±0.026 | 0.035±0.025 |
| Cellulolysis | 0.095±0.026 | 0.030±0.019 | 0.025±0.011 | 0.075±0.030 |
| Reductive acetogenesis | 0.093±0.029 | 0.028±0.019 | 0.018±0.005 | 0.074±0.028 |
| Predatory or exoparasitic | 0.011±0.011 | 0.007±0.007 | 0.095±0.039 | 0.082±0.063 |
| Aromatic compound degradation | 0.012±0.011 | 0.026±0.015 | 0.065±0.013 | 0.088±0.039 |
| Sulfate respiration | 0.009±0.007 | 0.019±0.007 | 0.116±0.028 | 0.046±0.010 |
| Respiration of sulfur compounds | 0.009±0.007 | 0.019±0.007 | 0.116±0.028 | 0.046±0.010 |
| Nitrate denitrification | 0.004±0.005 | 0.007±0.002 | 0.130±0.038 | 0.014±0.007 |
| Nitrite denitrification | 0.004±0.005 | 0.007±0.002 | 0.130±0.038 | 0.014±0.007 |
| Nitrous oxide denitrification | 0.004±0.005 | 0.007±0.002 | 0.130±0.038 | 0.014±0.007 |
| Denitrification | 0.004±0.005 | 0.007±0.002 | 0.130±0.038 | 0.014±0.007 |
| Sulfite respiration | 0.005±0.007 | 0.014±0.010 | 0.084±0.031 | 0.028±0.015 |
| Chlorate reducers | 0.002±0.002 | 0.009±0.007 | 0.032±0.007 | 0.007±0.007 |
| Chitinolysis | 0.002±0.002 | 0.002±0.002 | 0.009±0.009 | 0.016±0.015 |
| Photoheterotrophy | 0.005±0.004 | 0.004±0.002 | 0.005±0.004 | 0.002±0.002 |
| Phototrophy | 0.005±0.004 | 0.004±0.002 | 0.005±0.004 | 0.002±0.002 |

The data was showed as average ± deviation (n=3).
