# Supplementary material for: Dynamic variation of bacterial community assemblage and functional profiles during rice straw degradation
Source: Front Microbiol. 2023 Apr 14;14:1173442. doi: 10.3389/fmicb.2023.1173442 (PMC10140369; doi:10.3389/fmicb.2023.1173442)
Supplement: Supplementary file 2 [file Table_2.DOCX]

Table S2 Characteristics of the isolated strains

| **Type** | **Strain ID** | **D/d** | **Taxonomy** | **Accession number** | **Similarity (%)** |
| --- | --- | --- | --- | --- | --- |
| Cellulose-degrading strain | X7H1 | 3.43 | *Klebsiella quasipneumoniae* | CP029597.1 | 99.02 |
|  | X7H4 | 1.59 | *Chitinophaga eiseniae* | NR_116796.1 | 98.85 |
|  | X7C5 | 1.88 | *Chitinophaga eiseniae* | NR_116796.1 | 99.14 |
|  | X15H1 | 3.89 | *Bacillus thuringiensis* | KU161299.1 | 99.76 |
|  | X15H2 | 4.06 | *Klebsiella pneumoniae* | MF767576.1 | 99.50 |
|  | X15C2 | 3.46 | *Bacillus cereus* | JF509359.1 | 99.79 |
|  | X15C3 | 3.74 | *Bacillus cereus* | KF835391.1 | 100.00 |
|  | X15C4 | 2.75 | *Bacillus licheniformis* | JX847111.1 | 98.06 |
|  | X60H1 | 3.80 | *Stenotrophomonas maltophilia* | DQ466570.1 | 99.65 |
|  | X60H3 | 5.56 | *Chitinophaga eiseniae* | NR_116796.1 | 99.07 |
|  | X60C2 | 1.77 | *Bacillus subtilis* | JQ904714.1 | 100.00 |
|  | X60C3 | 5.01 | *Sphingobacterium* sp. | KY767658.1 | 97.73 |
|  | X120H1 | 4.01 | *Bacillus licheniformis* | KX871899.1 | 100.00 |
|  | X120H3 | 3.22 | *Chitinophaga eiseniae* | NR_116796.1 | 98.96 |
|  | X120C1 | 1.25 | *Bacillus subtilis* | KP974276.1 | 98.82 |
|  | X120C2 | 4.53 | *Escherichia coli* | CP027701.1 | 98.95 |
| Lignin-degrading strain | M7H2 | 1.53 | *Escherichia coli* | CP030331.1 | 99.44 |
|  | M7C1 | 2.15 | *Raoultella planticola* | CP023877.1 | 99.58 |
|  | M15H2 | 3.24 | *Klebsiella variicola* | HQ259961.1 | 99.51 |
|  | M60H3 | 2.94 | *Bacillus megaterium* | MF614908.1 | 99.72 |
|  | M60C2 | 4.56 | *Enterobacter ludwigii* | FJ462703.1 | 99.16 |
|  | M120H2 | 4.90 | *Pseudomonas monteilii* | MF045811.1 | 99.72 |
|  | M15C3 | 5.78 | *Klebsiella quasipneumoniae* | CP029597.1 | 99.02 |

D/d indicates the ratio of the diameter of the transparent circle (D) to the diameter of the colony (d).

The taxonomy of the strain was determined by BLASTn in the National Center for Biotechnology Information.
